# Factors Predicting Depression across Multiple Domains in a National Longitudinal Sample of Canadian Youth

**DOI:** 10.1007/s10802-014-9940-3

**Published:** 2014-09-21

**Authors:** Sherry Bellamy, Cindy Hardy

**Affiliations:** 1 School of Health Sciences, University of Northern British Columbia, 3333 University Way, Prince George, BC V2N 4Z9 Canada; 2 Department of Psychology, University of Northern British Columbia, 3333 University Way, Prince George, BC V2N 4Z9 Canada

**Keywords:** Longitudinal, Depression, Risk, Population sample

## Abstract

This prospective longitudinal study aimed to investigate the strength and relative importance of multiple predictors of depression in youth aged 16 to 20 years. Data were drawn from Statistics Canada’s National Longitudinal Survey of Children and Youth (Statistics Canada 2007a, b). Hierarchical regressions were conducted separately by child gender (*N* = 796 boys; *N* = 919 girls) for two overlapping samples: mixed parent–child dyads (e.g., biological mothers, fathers and other caregivers; *N* = 1,715) and a subsample containing only biological mother-child dyads (*N* = 1,425). Parent-reported data were used from Cycle 1 when the children were aged 4 to 8 years. Parent and child-reported data were used from Cycle 4 when children were aged 10 to 14 years. The outcome measure of depressive symptoms was taken from Cycle 7 when the youth were aged 16 to 20 years. Adolescents reported more depression symptoms than young adults and girls reported more than boys. For boys, higher anxiety/depression scores at ages 4 to 8 years and 10 to 14 years, along with lower self-esteem at 10 to 14 years, predicted higher depression scores. Girls’ depression was predicted by loss of a parent by ages 4 to 8 years and higher self-reported anxiety/depression and aggression at ages 10 to 14 years. Among biological mother-child dyads, maternal depression reported by mother when child was aged 4 to 8 years and 10 to 14 years significantly predicted depression for girls. At 10 to 14 years, child-reported lower parental monitoring (girls only) and greater parental rejection (boys and girls) predicted depression at ages 16 to 20 years.

## Introduction

Depression is a common mental illness that affects people at all stages of life, with peak prevalence in late adolescence and early adulthood (Patten et al. 2006). Much evidence from the interdisciplinary study of depression indicates that depression has genetic and environmental risk factors and that risk for depression begins to accumulate very early in an individual’s life (Brown et al. 1999; Kosterman et al. 2010). Depression was recently recognized as a leading cause of disability worldwide (World Health 2012). Longitudinal research examining risk for depression in diverse groups of children and youth increases our understanding of strategies for prevention, detection, and early intervention.

The aim of the present study is to contribute to and expand on existing research focused on investigating the effects of multiple childhood factors thought to increase risk for depression in young adulthood. In this longitudinal prospective study, reports from Canadian parents and children on behavioral and psychological characteristics of the child, parenting qualities, and peer relationships are investigated to determine their effects on the outcome of depression in adolescence and young adulthood.

Numerous mechanisms are involved in the development of depression with dynamic processes interacting; across individuals and across time, multiple pathways lead to depression through various combinations of direct and indirect effects (Rutter and Sroufe 2000). Early risk factors have been of great interest to those studying the development and etiology of depression because modification of risk factors is seen as means of prevention and/or early intervention. Knowing which risk and protective factors to target and when is essential for effective prevention (Kosterman et al. 2010). Methodologically, longitudinal investigations of nationally representative samples of children are invaluable as they address issues of timing and content across time and across diverse family and community contexts (Kosterman et al. 2010). Following a group of children longitudinally, as the children grow and develop, offers the best evidence concerning causes and continuity of specific disorders and associated risk factors (Costello et al. 2003).

Diverse risk factors across biopsychosocial and developmental domains contribute to depression. In the present study, the selection of predictor variables included in our analysis was guided by a comprehensive literature review of childhood risk factors for depression, and limited to measures available in the NLSCY. The extensive literature review is summarized briefly in the following paragraphs. Readers may contact the authors to request the complete literature review.

Girls and women experience higher rates of depression than do boys and men (Galambos et al. 2004), with the lifetime rate of depression estimated to be 1.5 to 3 times higher for women compared to men (Goodman and Tully 2008). Men and women experience unique and shared risk factors for depression (Kendler et al. 2002; Kendler et al. 2006). Depressive symptoms increase with age in childhood and adolescence; the increase is especially marked for girls aged 12 to 14 years (Galambos et al. 2004; Nolen-Hoeksema et al. 1991; Petersen et al. 1991). Boys also experience increased rates of depressive symptoms through adolescence, but to a lesser extent (Petersen et al. 1991).

Depression in youth often co-occurs with other disorders and the occurrence of “pure” depression is the exception rather than the rule (Angold et al. 1999; Bhardwaj and Goodyer 2009; Gallerani et al. 2010). The temporal sequence of onset of symptoms of depression and other disorders such as conduct disorder (Ezpeleta et al. 2006), oppositional defiant disorder (Nock et al. 2007), attention deficit hyperactivity disorder (Elia et al. 2008), and anxiety (Seligman and Ollendick 1998) suggest that symptoms associated with these disorders may be predictive of depression (Colletti et al. 2009).

Depression shows a tendency to aggregate in families (McGuffin and Katz 1989) and children of depressed parents experience higher rates of depression than children of non-depressed parents (Goodman and Tully 2008). First degree relatives of depressed probands experience higher rates of depression compared to control participants (Sullivan et al. 2000). Maternal depression is a robust predictor, more predictive than paternal depression, of depression and anxiety in children (Connell and Goodman 2002).

A variety of parent–child relationship factors predict the development of depression in children. Parental absence due to a variety of reasons is a risk factor for depression in children (Amato 1991). Loss of a parent through death (Cerel et al. 2006) and divorce is associated with higher rates of depression in children (Hoyt et al. 1990). Children who report that their parents use techniques such as psychological control, guilt induction and love withdrawal experience increased risk of depression (Barber 1996). Higher levels of parental control and parental rejection are associated with increased child depression (Garber et al. 2009b).

Youth who experience the social-behavioral deficits that characterize depression tend to engage in behaviours that create problems in their peer and family relationships, which can in turn exacerbate depressive symptoms and heighten the risk for further depressive episodes (Rudolph et al. 2008). Depressed youth often encounter conflict and rejection in their relationships and are perceived by themselves and others as having significantly impaired social skills (Rudolph et al. 2008). Low self-esteem in adolescence has been associated with greater risk for depression in young adulthood for both women and men (e.g. Boden et al. 2008; Orth et al. 2008).

Specific research questions investigated in this study were (1) Can depression in adolescence and young adulthood be predicted by specific social and psychological factors in a population sample of children? (2) Is there a change in the magnitude of effect of predictors in an exclusively biological mother and child subsample? (3) Are there gender differences in specific risk factors for depression?

## Methods

### The National Longitudinal Survey of Children and Youth (NLSCY)

The NLSCY is a longitudinal probabilistic survey designed to follow and monitor the development of a nationally representative sample of children across Canada’s 10 provinces. The NLSCY began in 1994/95 with subsequent data collection occurring every two years (Statistics Canada 2007a, b). The current research is based on data collected from the original cohort of children during Cycles 1 (1994/95), 4 (2000/01) and 7 (2006/07). The original cohort aged 0 to 11 years consisted of 22,831 respondents with a response rate of 86.5 %. The cumulative response rate for the longitudinal cohort was 67.8 % in Cycle 4 and 56.6 % in Cycle 7 (Statistics Canada 2007b).

At Cycle 1, the NLSCY sample is representative of Canadian children living in private family homes in the 10 provinces. Excluded are residents of the northern territories, families in the Armed Forces, and people living in institutions, Indian Reserves, and remote regions of the country. The sample was stratified by province and was large enough to yield reliable estimates for each age cohort (i.e., birth to 11 months, 1, 2–3, 4–5, 6–7, 8–9, and 10–11 years). Data collection for the NLSCY occurred with paper questionnaires, computer-assisted interviewing and computer assisted telephone interviewing. Measures were collected from the child once old enough and the person most knowledgeable (PMK) about the child. In Cycle 1, the PMK was the biological mother for 89.9 % of the sample. The PMK was the father, not specified if biological or otherwise, for 8.2 % of the sample with the remaining respondents consisting of step, adoptive, foster or other caregivers who were not a parent (Statistics Canada 2007a). Self-completed questionnaires for children aged 10 to 17 years focused on topics tailored to each age group (Statistics Canada 2007b). Measurement of similar constructs over time was accomplished by tailoring measurement strategies to several age groupings of the children (e.g., reliance on PMK report at Cycle 1 when child was 4 to 8 years of age and child self-report at Cycle 4 when child was 10 to 14 years of age).

### The Present Study

#### Participants

A longitudinal subsample (*n* = 1,715) was selected for analysis. Criteria for inclusion were that the target child was between the ages of 4 and 8 years in Cycle 1 and data were available for all measures used in the present study. This mixed sample of biological mothers, fathers and other caregivers is referred to as the mixed sample. The subsample of biological mother-child dyads (*n* = 1,425) included only those cases where the PMK at Cycle 1 and Cycle 4 was the target child’s biological mother. This sample is referred to as the biological mother subsample.

#### Sample Characteristics and Representativeness

The NLSCY sample included in the present study is described in Table 1. We examined variables central to this study, that is, child gender, child age, marital status of the PMK and depression scores of the PMK at Cycle 1. These variables were examined for differences between the sample retained for analyses and NLSCY participants of the same age who were excluded due to drop out or missing data. Children in the retained sample were more likely to be female and were slightly older, with proportionately more children who were 7 and 8 years old at Cycle 1. There were no significant differences in Cycle 1 PMK depression scores between the retained sample and respondents who did not meet inclusion criteria. Analysis of PMK marital status indicated PMKs who were retained in the study were more likely to be living with a partner either by marriage or co-habiting. Caution should be used when generalizing results to the population as the sample retained for analysis consists of an overrepresentation of girls, older children, and married or co-habiting PMKs, relative to the Canadian population of 1994/95.

**Table 1:** Child age, child gender, and PMK marital status for drop-out/missing and retained samples

	Drop-Out/Missing		Retained	
Characteristics	Proportions	95 % CI	Proportions	95 % CI
Child Age (years)				
4	21.70 %	[21.06, 22.35]	15.96 %	[13.45, 18.47]
5	20.79 %	[20.10, 21.48]	18.45 %	[15.68, 21.22]
6	20.17 %	[19.52, 20.82]	17.57 %	[15.01, 20.13]
7	18.67 %	[18.01, 19.34]	23.28 %	[20.57, 25.99]
8	18.66 %	[17.95, 19.38]	24.74 %	[21.88, 27.60]
Child Gender				
Boys	52.55 %	[51.7, 53.4]	45.7 %	[42.35, 49.05]
Girls	47.45 %	[46.6, 48.3]	54.3 %	[50.95, 57.65]
PMK Marital Status				
With Partner	83.38 %	[81.75, 85.01]	86.86 %	[83.98, 89.74]
Without Partner	16.62 %	[14.99, 18.25]	13.14 %	[10.26, 16.02]

*Note*. Proportions are shown for categorical variables. *CI*, confidence interval. Retained respondents had data on all predictor variables and the dependent variable (*n* = 1,715). Drop-out/ missing respondents are those who dropped out of the study after Cycle 1 or remained in the study but did not have data on all predictor variables or the dependent variable (*n* = 7,140)

### Bootstrapping Approach to Variance Estimation

To avoid publication of misleading results from complex surveys, Statistics Canada recommends researchers use the bootstrapping approach to variance estimation in order to account for design effects such as sample clustering and stratification (Statistics Canada 2007b). In order to estimate variance correctly, design effects such as sample stratification and clustering need to be accounted for (Roberts et al. 1999). Bootstrapping is a replicate-based technique in which repeated random samples are drawn from the original sample with replacement to estimate characteristics of the population (Fox 1997). Bootstrapping is used to estimate the error associated with the population parameter (Phillips 2004). For further information on the NLSCY the authors refer the reader to Statistics Canada documentation (Statistics Canada 2007b). For the present study, WesVar 4.3 was used to calculate variance estimates with a set of 1,000 bootstrap weights provided by Statistics Canada. Tests of significance and confidence intervals are reported based on results of bootstrapped estimation.

### Data Analysis

Prior to analysis, data were screened to ensure they met the distribution assumptions underlying inferential statistics. Screening revealed that variables were not normally distributed. For this reason, hierarchical regression analysis was chosen over structural equation modeling (SEM). It is advisable to use regression in cases where data distributions do not meet requirements of SEM as regression has established methods for identifying and handling assumption violations (Gefen, Straub, and Boudreau 2000). In this study, several variables including the dependent variable had to be transformed due to significant non-normality (i.e., skew). The outcome measure, self-reported youth depression at Cycle 7, was positively skewed; a square-root transformation resulted in acceptable normality. Logarithmic transformation was performed for the PMK (parent) depression scales for Cycle 1 and Cycle 4 as well as for the PMK-rated child anxiety and depression scale for Cycle 1 due to positive skew. Logarithmic transformation was performed for the aggression scores for Cycle 1 and Cycle 4. The friends and self-esteem scales were negatively skewed and were reflected and logarithmic transformed. Square-root transformations were conducted for hyperactivity scores for Cycle 1 and child-rated anxiety and depression scores for Cycle 4. Lastly, child-rated parental consistency for Cycle 1 and parental rejection scores for Cycle 4 were moderately negatively skewed and were reflected and square-root transformed. Transformations resulted in distributions suitable for regression analyses.

Hierarchical regression analyses were performed to examine the relationship between the dependent variable depression for youth aged 16 to 20 years and a set of independent variables measured when the children were aged 4 to 8 years and 10 to 14 years. The step by step analyses in hierarchical regression allows for the determination of the importance of predictor variables’ effects on the dependent variable over and above the effect of variables entered into the model first. The procedure and number of steps is determined according to the hypothesis being tested. In the current investigation, age was entered first. Cycle 1 predictor variables measured when the children were aged 4 to 8 years were entered at step 2 and Cycle 4 predictor variables measured when the children were aged 10 to 14 years were entered in the third step of the model. The same process was used for the mixed sample and biological subsample. Regressions were conducted separately by gender in order to determine potential differences in the effects of predictors of depression for boys and girls and because boys and girls differed on average depression scores at ages 16 to 20 years.

### Measures

Internal consistency for scaled measures was acceptable (Cronbach’s alpha greater than 0.60) with the exception of Parental Monitoring and Parental Consistency (see Statistics Canada 2007b, for more detailed information).

The outcome measure of depression for youth at 16 to 20 years of age and PMK depression was self-reported using a modified version (Statistics Canada 2007b) of the Center for Epidemiological Studies Depression Scale developed by Radloff (1977). The CES-D has high reliability in population samples (Scott and Melin 1998) and is a valid measure of depression in adolescent samples (Mojarrad and Lennings 2002). For the NLSCY, Statistics Canada removed 8 items from the CES-D to create a 12 item questionnaire. Participants indicated how well the statements described their mood/behavior over the past week. For specific scale items see Statistics Canada (2007b). The shortened depression scale used in the NLSCY was found to have good internal consistency and acceptable discriminative validity in a sample of 12,990 Canadian adolescents (Poulin et al. 2005).

## Results

### Regression Analysis

Results for regression analyses predicting youth depression are presented separately for boys and girls, first for the mixed sample followed by the biological mother subsample. The biological mother subsample is of particular interest because it provides evidence about the genetic transmission of depression. If risk factors associated with childhood depression have genetic underpinnings, we would expect a greater magnitude of effect when PMKs are biological parents (in this case, mothers) relative to the sample of mixed PMKs. If there is little genetic contribution we would expect to see similar or lesser magnitude of effects. We used only mothers for the biological parent subsample because the sample of biological father PMKs was too small to analyze. Regression results are presented in Table 2; table entries reflect the effect size attributable to each predictor variable when other variables in the equation are held constant (i.e., squared semi-partial correlation).

**Table 2:** Results for hierarchical multiple regressions predicting youth depression (sr^2^)

	Mixed Sample		Biological Mother Subsample	
Predictor Variable	Boys	Girls	Boys	Girls
Step 1				
Age	−0.34***	−0.20***	−0.34***	−0.25***
Step 2				
Age	−0.32***	−0.20***	−0.32***	−0.25***
C1 PMK Depression	−0.02	0.10*	−0.04	0.10*
C1 Hyperactivity	−0.05	0.02	−0.06	−0.02
C1 Anxiety/Depression	0.11**	0.02	0.13**	0.06
C1 Aggression	0.05	−0.01	0.06	−0.01
C1 Positive Interaction	−0.02	−0.03	−0.01	−0.06
C1 Parental Consistency	0.01	−0.04	0.01	−0.01
CI Hostile Punitive	−0.05	0.02	−0.05	0.01
C1 Biological Parent	0.04	0.12**	0.03	0.13**
Step 3				
Age	−0.23***	−0.24***	−0.27***	−0.29***
C1 PMK Depression	−0.01	0.06	−0.02	0.06*
C1 Hyperactivity	−0.07	−0.01	−0.07	−0.04
C1 Anxiety/Depression	0.09*	0.02	0.11**	0.07
C1 Aggression	0.04	−0.02	0.05	−0.02
C1 Positive Interaction	−0.03	−0.02	−0.01	−0.04
C1 Parental Consistency	0.01	−0.04	0.01	0.01
CI Hostile Punitive	−0.05	−0.01	−0.06	−0.03
C1 Biological Parent	0.03	0.08	0.04	0.09
C4 PMK Depression	0.01	0.09	−0.01	0.12**
C4 Friends	0.01	0.01	0.02	0.03
C4 General Self	0.10*	0.04	0.12*	0.04
C4 Anxiety/Depression	0.21***	0.10**	0.20***	0.14**
C4 Hyperactivity	−0.03	−0.03	−0.04	−0.06
C4 Aggression/CD	−0.01	0.07**	−0.04	0.06*
C4 Parental Nurturance	−0.03	−0.04	−0.02	−0.05
C4 Parental Monitoring	0.05	0.08	0.02	0.12**
C4 Parental Rejection	0.06	−0.08	0.10*	0.09*
C4 Biological Parent	−0.01	0.01	−0.03	−0.02
R-Squared for Model	0.24***	0.15	0.25**	0.21***

*Note. C1*, Cycle 1; *C4*, Cycle 4. R^2^ for each step were not available from the bootstrapping software used to estimate *sr* ^*2*^

* *p* < 0.05., ** *p* < 0.01., *** *p* < 0.001

#### Boys, Mixed Sample

For boys (*n* = 796) in the sample of mixed PMKs, 24 % of the overall variance in depression was explained by the predictors in the model. Age was entered first and significantly predicted depression, *β* = −0.34, *p* = 0.001, 95 % CI [−0.35, −0.19], indicating the adolescents in the sample scored higher in depression compared to the young adults. Cycle 1 predictors were entered in the second step and as a group significantly predicted depression, although only one variable was significant on its own. Higher levels of parent-reported child anxiety and depression scores at ages 4 to 8 years, *β* = 0.13, *p* = 0.008, 95 % CI [0.14, 0.91] predicted depression. Cycle 4 variables were entered in the third step and two variables significantly predicted depression. Lower levels of self-reported self-esteem at ages 10 to 14 years, (*β* = 0.13, *p* = 0.044), 95 % CI [0.01, 0.96], and higher levels of self-reported anxiety and depression scores at ages 10 to 14 years, *β* = 0.26, *p* < 0.001, 95 % CI [0.18, 0.46], predicted depression when boys were aged 16 to 20 years.

#### Girls, Mixed Sample

For girls in the sample with mixed PMKs (*n* = 919), the total variance in depression scores explained by the model was 15 %. Age predicted depression (*β* = −0.20, *p* = 0.001), 95 % CI [−0.23, −0.09] indicating adolescent girls reported higher rates of depression compared to young adults. Significant predictors in the second step of the model included higher levels of self-reported PMK depression scores (*β* = 0.11, *p* = 0.025), 95 % CI [0.04, 0.59], and the loss of a biological parent from the home (*β* = 0.12, *p* = 0.008), 95 % CI [0.09, 0.62]. Significant Cycle 4 predictors added in step three included higher self-reported anxiety and depression scores (*β* = 0.13, *p* = 0.014), 95 % CI [0.03, 0.3], and higher self-rated aggression and conduct disorder scores (*β* = 0.04, *p* = 0.018), 95 % CI [0.01, 0.07] predicted depression at ages 16 to 20 years.

#### Boys, Biological Mother Subsample

For boys in the biological mother-child subsample (*n* = 686) the predictor variables explained 25 % of variance in depression scores with only slightly more variance explained than in the mixed sample (24 %). The overall pattern of findings in the biological subsample was the same as for the mixed sample, with one addition. In the biological subsample, higher levels of parental rejection reported by boys at ages 10 to 14 years also predicted later depression (*β* = 0.12, *p* = 0.02), 95 % CI [0.01, 0.05].

#### Girls, Biological Mother Subsample

For girls, (*n* = 797) the predictor variables explained 21 % of the overall variance in depression scores. This is a large increment in explained variance relative to that explained in the mixed sample regression (15 %) and additional predictors of depression were significant. Higher maternal depression scores emerged as a significant predictor for depression in girls at both Cycle 1 (*β* = 0.12, *p* = 0.03), 95 % CI [0.03, 0.64] and Cycle 4 (*β* = 0.14, *p* = 0.015), 95 % CI [0.07, 0.67]. Additionally, higher self-rated parental rejection scores (*β* = 0.02, *p* = 0.047), 95 % CI [0.00, 0.05] and lower self-rated parental monitoring scores (*β* = 0.14, *p* = 0.002), 95 % CI [0.09, 0.40] increased girls risk for depression.

## Discussion

The goal of the study was to investigate the effects of a wide range of risk factors of depression in a longitudinal sample of Canadian children. The authors hypothesized depression in adolescents and young adults aged 16 to 20 years could be predicted by specific social and psychological factors across childhood. The authors also expected to find differences and similarities in the effects of predictors for depression according to gender of child; and an increase in the magnitude of effects for the biological mother-child subsample relative to the mixed sample. The hypotheses were supported; depression was predicted by behavioral, social and psychological factors measured 8 years and 4 years prior to the outcome of depression measured at aged 16 to 20 years. Gender differences and similarities were found, and the biological mother and child subsample revealed stronger effects for some predictors, and for girls greater predictive power overall, relative to the mixed sample.

### Gender Differences and Similarities in the Pattern of Risk Factors for Depression

Age predicted depression for both genders. Depression scores were higher in the lower age ranges of 16 to 20 years. Past longitudinal research has demonstrated similar effects of age with the highest incidence and greatest increase in new cases of depression occurring for both boys and girls between the ages of 15 to 18 years (Hankin et al. 1998). These findings indicate mid to late adolescence may be a particularly vulnerable period for the development of depression in both boys and girls.

Higher levels of depression and anxiety at younger ages predicted later depression for both genders. However, this measure emerged as a stronger predictor for boys compared to girls. For boys, parent-reported anxiety and depression at ages 4 to 8 years and self-reported anxiety and depression at ages 10 to 14 years predicted depression at ages 16 to 20 years. For girls, self-reported anxiety and depression at ages 10 to 14 years predicted later depression while parent reports of anxiety and depression at ages 4 to 8 years did not. Considered in light of evidence that, in comparison to women, men with generalized anxiety had a greater risk for depressive episodes especially when coupled with stressful life events (Hettema et al. 2006), these findings indicate anxiety may be a greater liability for later depression in boys compared to girls. These results also provide evidence for continuity of depressive symptoms over time with early symptoms in childhood continuing into adolescence and adulthood.

Lower levels of self-reported self-esteem at ages 10 to 14 years predicted depression at ages 16 to 20 years for boys but not girls. While several studies have shown self-esteem predicts depression in both genders, (e.g. Boden et al. 2008; Orth, et al. 2008), there is some evidence to suggest boys with low self-esteem may be particularity vulnerable to depression. In two separate investigations of multiple risk factors for depression, Kendler and colleagues found low self-esteem predicted depression in men but not in women (Kendler et al. 2002, 2006). The current study provides prospective evidence based on a population sample linking low self-esteem to depression in boys but not girls.

In considering the causal effect of low self-esteem, some researchers argue low self-esteem should be viewed as a risk marker that reflects other factors related to the development of depression. For example Boden et al. (2008) found low self-esteem at age 15 years predicted later depression, however upon further investigation the authors found low self-esteem tended to be more common in individuals who had lower IQ levels, previous mental health problems, higher levels of neuroticism, lower socioeconomic status, increased family dysfunction and childhood adversities such as poor parental bonding or physical and sexual abuse.

It is also worth noting that in studies investigating self-esteem in adolescence, boys tend to score higher on measures of self-esteem compared to girls (e.g., Boden et al. 2008; Derdikman-Eiron et al. 2011; Orth et al. 2008). Speculation on the current findings in light of past research suggests that because boys tend to score higher on self-esteem measures, boys who score low on self-esteem may be especially vulnerable due to exposure to other risk factors for depression. However, further research is needed to clarify the nature of the association between low self-esteem and depression in boys.

The loss of a parent by the ages of 4 to 8 years predicted depression at ages 16 to 20 years for girls but not for boys. The current study did not differentiate between death, separation, or divorce as the cause for parental loss, in line with past research indicating that parental absence due to a variety of reasons has been associated with the development of depression (e.g. Amato 1991). Several explanations are offered to explain the link between parental absence in childhood and subsequent depression. Compared to intact families, divorced or separated families more often have higher levels of parental depression, lower levels of family function and lower marital satisfaction (Strohschein 2005). The effects of parental divorce have been found to differ between genders in respect to the development of depression with risk increasing for girls but not for boys (Oldehinkel et al. 2008). Moreover, McCabe (1997) found young women whose parents had divorced reported higher levels of depression compared to young men from divorced families.

The present study found higher levels of self-reported child aggression and conduct disorder at ages 10 to 14 years predicted depression for girls but not for boys. These results are in line with prior longitudinal studies that have found links between aggression and conduct disorder for girls but not boys. For example, Costello et al. (2003) found significant comorbidity between conduct disorder and depression for girls but not for boys. Further, Wiesner and Kim (2006) found links between developmental trajectories of delinquent behavior and depression were stronger for girls than for boys. The current study provides further evidence the behavioral dimension of aggression is an important predictor of depression for girls but not boys.

### Increased Magnitude of Effects in the Biological Mother-Child Subsample

PMK and maternal depression when the children were aged 4 to 8 years predicted depression for girls but not for boys. As hypothesized, maternal depression in the biological mother-child subsample yielded stronger effects compared to the mixed sample of PMKs, predicting depression for girls at ages 4 to 8 years and 10 to 14 years. In a meta-analysis designed to examine the magnitude of effects between maternal and paternal psychopathology and internalizing and externalizing disorders in children, Connell and Goodman (2002) expected to find stronger effects in studies that focused on exclusively biological parents and their children given the significant heritability of psychopathology. No difference in magnitude of effect was found between studies indicating their sample was exclusively biological and studies using mixed samples or those that did not provide information on the biological status of the sample. However, caution was advised in the interpretation of results as the sub-samples were not found to be homogeneous. The present study provides evidence of stronger magnitude of effects of maternal depression in relation to the development of depression in girls in an exclusively biological mother sample. Given that a large portion of the mixed sample who were excluded from the biological mother and child analysis were fathers, the authors caution against interpreting the increase in magnitude of effects as being due to shared mother-child biology. It may also be due to the exclusion of fathers from the sample.

In the biological mother and child subsample child-rated parenting measures predicted depression. Girls who rated their parents lower on the monitoring scale at ages 10 to 14 years were at increased risk to develop depression at outcome. It is important to note parental monitoring is a bidirectional process occurring between children and parents and is affected by several aspects including child disclosure (Kerr and Stattin 2000; Stattin and Kerr 2000) and parental interest and efforts to know about the child’s activities, peers and whereabouts (Berg-Nielsen et al. 2003). The reciprocal nature of parental monitoring may explain why this measure is associated with depression for girls but not boys. Studies have shown girls tend to disclose more to parents than boys (e.g. Kerr and Stattin 2000); indicating girls may require closer relationships with parents. This conclusion is supported by the finding that, compared to boys, adolescent girls have been shown to demonstrate a higher proclivity for communion, expressed through connectedness (Davies and Lindsay 2004). The association between perceived levels of parental monitoring and increased risk for depression in girls may reflect low connectedness with parents and subsequent risk for depression.

Higher levels of child-reported parental rejection at ages 10 to 14 years predicted depression for both girls and boys in the biological mother and child subsample. These results are not surprising as the link between parental rejection and depression and anxiety in children and adolescents is well established (Magaro and Weisz 2006; Rapee 1997; van der Bruggen et al. 2010).

The significance of parental monitoring and parental rejection emerging as predictors of depression in the biological mother and child subsample but not the mixed sample is open to speculation. It is possible the stronger effects of the parenting measures in the biological mother and child subsample may be due to genetic mediation of environmental influences. According to Rutter et al. (2001) research has consistently demonstrated associations between family characteristics and child behaviors are stronger in biological families, indicating many environmental measures have a degree of genetic mediation. The implication is the child’s interpretation of parental characteristics may reflect a reciprocal interaction between environment and genetics as the environment is influenced by genetics. Research has found perceptions of social support, life satisfaction and measures of depression receive nearly equal influence by environment and genetics (Bergeman et al. 1991). The parental effects found in this study may involve interplay between genetics and environment as it is possible children prone to depression are more likely to rate their parents as low in monitoring and high in rejection.

Future research should seek to further investigate potential differences in effects of predictors of depression between biologically and nonbiologically related parents and children. Many studies include both biological and nonbiological parents in their studies or fail to report the biological status of the parents and children in their sample (Connell and Goodman 2002). The inclusion of information regarding the biological status of parents and children offers an important contribution to the investigation of risk of psychopathology in childhood. Further, it will be important to consider the timing and severity of maternal depression as a risk factor for the development of depression in offspring. While there are challenges to including a full history of depressive symptoms in mothers, especially with large samples, the inclusion of this information is invaluable for determining the dose–response relationship between maternal depression and child outcomes.

### Limitations

Although longitudinal studies offer important information in terms of understanding risk processes (Rutter and Sroufe 2000) the generalization of results is limited due to sample attrition and changes occurring in the population over time (der Kamp et al. 1998).

Because the study investigated depression it was important to know whether attrition was a function of depressive symptoms in the parent. The missing data analysis found no significant differences in PMK depression symptoms between participants lost at follow up and participants who remained in the study. However, the missing data analysis of the current study indicated there were proportionately more dual parent families in the sample remaining in the study than in the sample lost to attrition. This is an important factor to consider as a prior longitudinal study investigating a large community sample found mothers who were single or cohabiting with a partner that was not a biological parent of the child were more than twice as likely to report elevated depression symptoms compared to mothers parenting with their child’s biological father (O’Connor et al. 1998).

It is also important to consider the absence of certain measures in the study; data were not available for the occurrence of sexual abuse, physical abuse or neglect. It is well established that childhood abuse and neglect is associated with increased risk for the development of psychopathology (e.g. Brown et al. 1999; Putnam 2003; Silverman et al. 1996). Additionally, the measure of depression used in the NLSCY was restricted to a measure of depression symptoms over the past week and did not provide information on the history of depression symptoms in the individual. This is an important limitation as depression is episodic and recurrent and a snapshot in time does not likely capture the full extent of illness in an individual. Further, the design of this particular study did not include measures of parental depression before the children were aged 4 to 8 years of age. Research has shown that children exposed to maternal depression at younger ages are vulnerable to internalizing and externalizing disorders (Connell and Goodman 2002) as well as psychopathology and emotional disorders (Goodman 2007; Goodman et al. 2011; Goodman and Gotlib 1999).

All measures used in the NLSCY were shortened versions of the original measures. Most NLSCY instruments yielded adequate reliability measures when analyzed with the NLSCY sample, however, the scales for childhood aggression, parental rejection, and parental monitoring yielded somewhat lower measures of reliability. Finally, while Statistics Canada’s recommendation to conduct bootstrapping analysis is beneficial in order to control for design effects, the software WesVar does not produce *R* ^2^ change values. The inclusion of R^2^ change values is often desirable as it represents the additional amount of variance explained in each step of the model.

### Implications

This study offers several important novel findings, as there are few longitudinal studies with genetically informed sample designs (meaning the biological status of the mother is accounted for) investigating gender differences in childhood risk factors for the development of depression. The findings inform prevention efforts with evidence of the relative importance of specific predictors across development for boys and girls. Researchers in the field of prevention have expressed the need for studies to inform prevention efforts so they may design targeted, specific and personalized intervention (Reynolds 2009). The findings indicate that boys and girls may benefit from early interventions designed to detect and reduce childhood anxiety and depression. Boys may benefit from interventions designed to increase self-esteem and reduce anxiety whereas girls may benefit from interventions designed to address parental loss due to death, divorce, and other causes. Family interventions designed to reduce parental depression and to strengthen parent–child relationships may reduce the child’s risk for depression. These implications are important for public health as research shows that prevention efforts for at risk youth are effective in preventing depression (Garber et al. 2009a).

The study identified several factors that increase risk of the development of depression symptoms in both boys and girls. Overall, girls have a greater risk of developing depression with multiple parental, behavioral and psychological risk factors. Specifically, in biological mother-child dyads, maternal depression places girls at higher risk to develop depression. Negative parenting characteristics and losing a parent place girls at higher risk for later depression. Childhood symptoms of anxiety and depression place children, especially boys, at risk for depression in late adolescence. In addition, boys with lower levels of self-esteem and perceptions of parental rejection are at increased risk for depression.
